# A Critical Assessment of 60 Years of Maize Intragenic Recombination

**DOI:** 10.3389/fpls.2018.01560

**Published:** 2018-10-29

**Authors:** Ron J. Okagaki, Stefanie Dukowic-Schulze, William B. Eggleston, Gary J. Muehlbauer

**Affiliations:** ^1^Department of Agronomy and Plant Genetics, University of Minnesota, St. Paul, MN, United States; ^2^Department of Horticultural Science, University of Minnesota, St. Paul, MN, United States; ^3^Department of Biology, Virginia Commonwealth University, St. Paul, MN, United States; ^4^Department of Plant and Microbial Biology, University of Minnesota, St. Paul, MN, United States

**Keywords:** recombination, hotspots, intragenic, polarity, double-strand breaks, maize

## Abstract

Until the mid-1950s, it was believed that genetic crossovers did not occur within genes. Crossovers occurred between genes, the “beads on a string” model. Then in 1956, Seymour Benzer published his classic paper describing crossing over within a gene, intragenic recombination. This result from a bacteriophage gene prompted Oliver Nelson to study intragenic recombination in the maize *Waxy* locus. His studies along with subsequent work by others working with maize and other organisms described the outcomes of intragenic recombination and provided some of the earliest evidence that genes, not intergenic regions, were recombination hotspots. High-throughput genotyping approaches have since replaced single gene intragenic studies for characterizing the outcomes of recombination. These large-scale studies confirm that genes, or more generally genic regions, are the most active recombinogenic regions, and suggested a pattern of crossovers similar to the budding yeast *Saccharomyces cerevisiae*. In *S. cerevisiae* recombination is initiated by double-strand breaks (DSBs) near transcription start sites (TSSs) of genes producing a polarity gradient where crossovers preferentially resolve at the 5′ end of genes. Intragenic studies in maize yielded less evidence for either polarity or for DSBs near TSSs initiating recombination and in certain respects resembled *Schizosaccharomyces pombe* or mouse. These different perspectives highlight the need to draw upon the strengths of different approaches and caution against relying on a single model system or approach for understanding recombination.

## Introduction

Recombination is the exchange of genetic information between chromosomes. Meiotic recombination is a major contributor to genetic diversity and facilitates selection by nature and breeders. A large share of our current understanding of recombination is based on work studying intragenic recombination (recombination within genes) in model fungal species, especially the budding yeast (*Saccharomyces cerevisiae*). Conclusions from genetic fungal studies have been supported by recent molecular and genomic approaches, providing a relatively detailed, although still incomplete, picture of recombination (reviewed in Keeney et al., 2014; Gray and Cohen, 2016). Beginning in the early 2000s, studies in the plant model system *Arabidopsis thaliana* have supported a similar picture of recombination (Wang and Copenhaver, 2018). Maize has been a genetic model organism since the early 1900s, and there is an extensive history of intragenic recombination studies in maize. Maize studies identified genes as recombination hotspots with crossovers distributed approximately evenly across many genes, which conflicts with the discrete hotspots and polarity found in *S. cerevisiae*. Our purpose here is to review the maize intragenic recombination work, and place this work in context with results from genomic studies of maize recombination and work in fungal and animal model systems.

**FIGURE 1:**
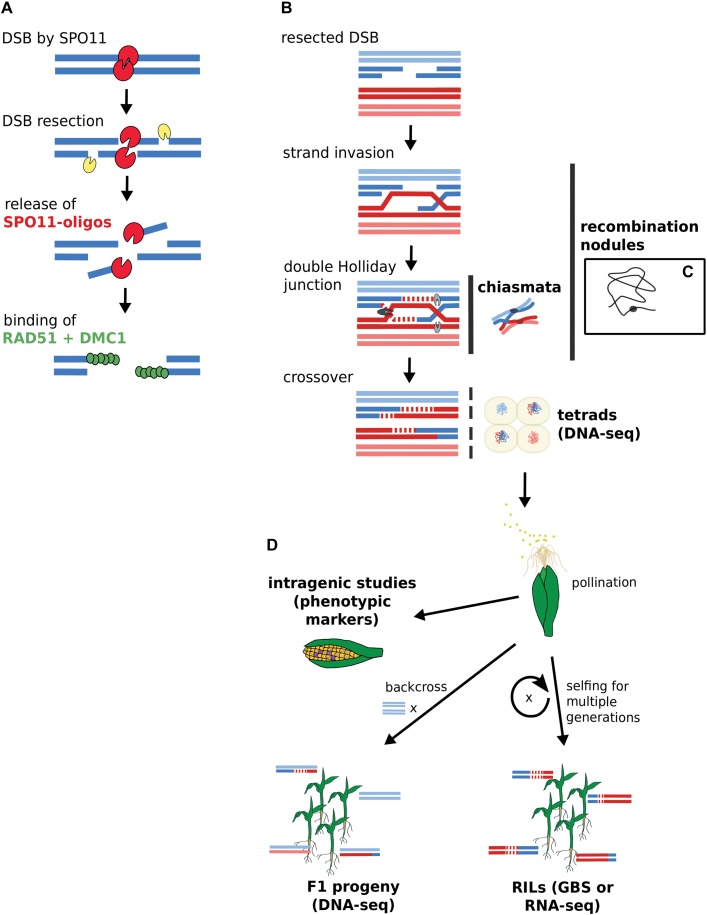
Acquisition of DSB and CO data by gene-scale and genome-scale approaches. **(A)** DSB generation by SPO11 with subsequent binding of RAD51 and DMC1. DSB data derives from SPO11-oligos or RAD51-bound fragments via ChIP-seq. **(B)** CO generation via double Holliday junction. **(C)** Chiasmata and recombination nodules are visible via microscopy. A single recombination nodule on a chromosome is illustrated. **(D)** Mapping recombinants. Sequencing approaches rely on isolated microspores from tetrads or progeny lines. Intragenic studies directly score recombination via visible kernel markers. Terms in bold indicate data source options. RILs, recombinant inbred lines; GBS, genotyping by sequencing.

To fully comprehend what intragenic as well as large-scale genomics studies can tell us about recombination, we recapitulate and reconcile knowledge from historic and more recent studies. Figure 1 depicts the approaches for gene-scale and genomic-scale to illustrate their data origins and differences. To facilitate a smooth and easy understanding of the information in this review, we first clarify the following terms which are frequently used:

• **“Recombination”** is a term used for mechanisms of somatic DNA repair as well as for exchange of genetic information during meiosis. While underlying mechanisms and involved proteins overlap, there are profound differences between somatic and meiotic recombination. Meiotic recombination can refer to **crossovers (COs)** which are due to exchange of whole chromosome parts, to **non-crossovers (NCOs)** which are locally restricted, or to both. Both COs and NCOs can result in local genetic changes via gene conversion.
• **“Gene conversion” (GC)** is the non-reciprocal transfer of information. Resolution of double Holliday structures into COs produces a gene conversion tract, as do pathways leading to NCOs. The latter is often used interchangeably with GC although it does not cover all GC instances.
• **“Intragenic recombination”** refers to both NCOs and COs within genes. Some intragenic recombination studies look at short regions containing several genes, albeit mostly with emphasis on the outcome within genes. These loci or genes are often recombination hotspots.
• **“Hotspots”** are genomic regions with elevated levels of recombination-related events, and can refer to the meiotic **double-strand breaks (DSBs)** initiating recombination, **COs** or **general recombination** including both COs and NCOs. There is no standard definition of hotspots regarding their strength or size. The amount of events distinguishing hotspots from cold regions is arbitrary, and the definition and identification depends strongly on the respective study.
• **“Polarity”** exists when there is a gradient of recombination, e.g., higher recombination rates at the 5′ or 3′ end of a gene. The 5′ end is defined here as the transcription start site (TSS), and the 3′ end as the transcription termination site (TTS). Other definitions have been used such as the promoter region for the 5′ end. However, in practice, COs are localized to intervals defined by available sequence polymorphisms which may not coincide with the defined 5′ and 3′ ends.

We have two goals for this review. First, we hope to show how the study of individual genes may influence our interpretation of genomic studies of recombination. Second, we describe characteristics in several model systems to illustrate the variation present in nature, and to argue that recombination in maize shares some, but not all, properties of each of these systems.

## A History of Maize Intragenic Recombination

The classical conception of genes posited that genes were indivisible units and recombination occurs between genes (reviewed in Green, 1955; Portin, 1993). Recombination within genes, intragenic recombination, was not believed to exist, especially since several apparent exceptions turned out to be recombination between duplicated gene copies in a complex locus. An alternative position was supported by several prominent geneticists who viewed genes as having multiple sites where crossing over could occur (Pontecorvo, 1955). Arthur Chovnick’s Perspectives article in Genetics provides a historical overview (Chovnick, 1989).

Today, Seymour Benzer’s papers demonstrating intragenic recombination in bacteriophage are often seen as the experimental work changing our understanding of recombination and genes (Benzer, 1955). At that time however, it was not clear. Several explanations for the contrasting results from bacteriophage versus *Drosophila melanogaster* and other familiar genetic systems were proposed (Green, 1955). One possibility was that recombination was different in bacteriophage and eukaryotes. Alternatively, detecting intragenic recombination might require screening very large populations.

Nelson (1957) proposed testing intragenic recombination at the maize *Waxy* (*Wx*) locus. *Wx* encodes a starch synthase required for amylose in the kernel endosperm and pollen. A recombination event between two mutant alleles would create a non-mutant *Wx* allele giving a revertant *Wx* pollen grain. Non-mutant *Wx* pollen contains a mixture of amylose and amylopectin starches and is stained a dark black by potassium iodine, while mutant *wx* pollen contains only amylose and stains reddish. This pollen phenotype is readily scored under a microscope and allows screening of very large numbers of meiotic products. Using this pollen assay, Nelson was able to detect intragenic recombination in a higher eukaryote (Nelson, 1959). A second study incorporated genetic markers flanking the *Wx* locus to connect recombination within the *Wx* locus with the exchange of flanking markers (Nelson, 1962).

**FIGURE 2:**
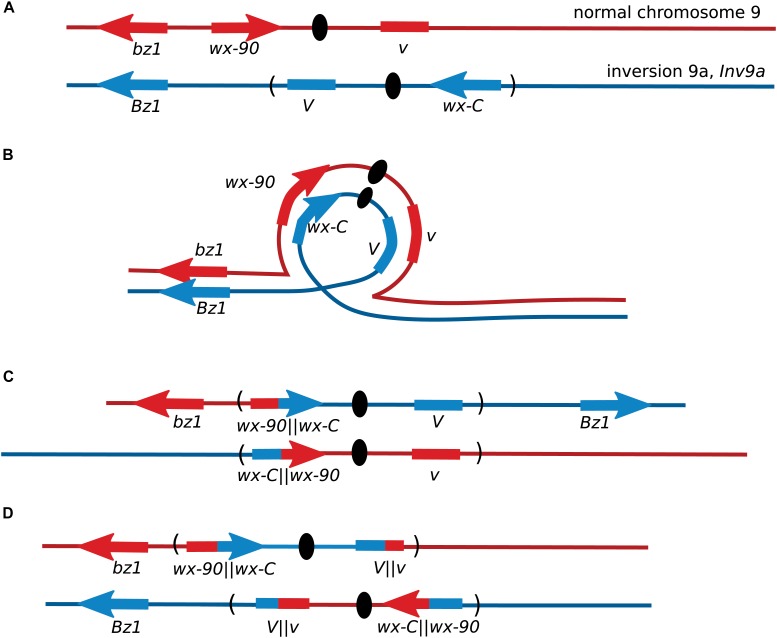
Recovery of maize *Wx* recombinants from an inversion heterozygote. **(A)** Locations of the *Wx* locus and the flanking markers *Bronze1* (*Bz1*) and *Virescent* (*V*) on the normal chromosome 9, and their locations on the pericentric inversion chromosome 9. The orientation of *V* is not known. **(B)** Chromosome pairing during meiosis. **(C)** Gametes from meiosis with crossovers within a pericentric inversion are generally inviable because one centromere carries with it both short arms and the other centromere carries both long arms. Non-crossover events may produce non-mutant *Wx* kernels. **(D)** Crossovers between *wx-C* and *wx-90* will produce inviable pollen unless there is a second crossover within the inversion. Most *Wx* revertant pollen will be from non-crossover events as double crossovers are rare in short genetic intervals. This illustration shows the second crossover occurring within *V*.

After the initial observation of intragenic recombination in maize, the *Wx* locus was used for further studies focusing on exploring the recombination process. Nelson’s studies provided early evidence for a non-crossover recombination pathway, by using lines where the *wx-C* allele was located inside a chromosome inversion or a complex chromosomal rearrangement (Nelson, 1975). Single-crossovers between these *wx-C* alleles on a rearranged chromosome and the *wx-90* allele on a normal chromosome produce inviable gametes unless there was a second crossover within the inversion (Figure 2). When both *wx-C* and *wx-90* were on normal chromosomes crossover events accounted for approximately 65% of the *Wx* revertants based on the segregation of flanking markers. There was crossing over between the flanking markers in 35% of the *Wx* revertants when *wx-C* was located within a pericentric inversion. A portion of these crossovers occurred outside of the inversion and accompanied a NCO event between the *wx* alleles. When *wx-C* was within a complex rearrangement the few revertants arose through non-crossover events.

The *Wx* pollen system was also used to explore whether the distance of a locus from the centromere altered recombination frequency (Yu and Peterson, 1973). Using chromosome translocation lines with *wx* alleles at different distances from the centromere they showed that distance of a locus from the centromere is correlated with recombination frequency. Other studies by Peterson examined the effects of chemical treatments on recombination and noted the effect of environment on recombination at the *Wx* locus (Sukhapinda and Peterson, 1980).

Pollen phenotyping procedures were developed for other genes to study intragenic recombination. Mike Freeling described an odd situation where *alcohol dehydrogenase1* (*adh1*) alleles derived from the same progenitor allele showed intragenic recombination, but *adh1* alleles derived from different progenitor alleles did not recombine (Freeling, 1978). One possibility suggested at the time was that local structural differences between progenitor alleles inhibited synapsis and recombination. This conjecture was supported by subsequent molecular findings of little similarity between regions flanking most, but not all, parental *Adh1* alleles (Johns et al., 1983; Sachs et al., 1986). In general, maize intragenic studies focused on genes with an easily scored phenotype, and most genes studied proved to be hotspots. A partial list of key results from intragenic studies is presented in Table 1.

**Table 1:** Landmark maize intragenic recombination studies.

Authors	Locus	Notable results
Stadler and Emmerling, 1956	*R1*	Recombination between genes in a complex locus
Nelson, 1959	*Wx*	Demonstration of intragenic recombination in a higher eukaryote
Nelson, 1968	*Wx*	Mapping transposable elements within a gene
Yu and Peterson, 1973	*Wx*	Physical distance from centromere affects recombination rates
Nelson, 1975	*Wx*	Evidence for crossover and non-crossover mechanisms
Freeling, 1978	*Adh1*	Role of flanking sequences in crossing-over
Dooner et al., 1985	*Bz1*	*Bz1* is a recombination hotspot, 100-fold higher than the genome average
Brown and Sundaresan, 1991	*A1*	Crossover hotspot in the 5′ coding region
Civardi et al., 1994	*A1-Sh2*	Fine mapping crossovers in a chromosomal region
Patterson et al., 1995	*B1*	Crossover hotspot in the 5′ coding region
Eggleston et al., 1995	*R1*	Unequal crossing-over within the complex *R-st* allele fractionates its epigenetic potential
Dooner and Martínez-Férez, 1997	*Bz1*	Recombination is uniform across the *Bz1* gene
Okagaki and Weil, 1997	*Wx*	The promoter region is not required for recombination at *Wx*
Yao et al., 2002	*A1-Sh2*	Hotspots and cold regions in a 140 kb region; a non-genic low-copy sequence can be a recombination hotspot
Dooner, 2002	*Bz1*	Low sequence diversity between alleles favors NCO pathway
Yao and Schnable, 2005	*A1-Sh2*	Sequence polymorphisms partially explain crossover distributions in hotspots
Yandeau-Nelson et al., 2006	*A1*	Choice of template in a tandem duplication, rare use of sister chromatid
Dooner and He, 2008	*Bz1* region	Impact of adjacent retrotransposon polymorphisms on recombination
Wang et al., 2011	278 kb region on chromosome 10	Gene density and recombination
Dooner and He, 2014	*Bz1*	NCO events show polarity at the 5′ and 3′ ends of the gene

With the cloning and sequencing of maize genes, it became possible to compare the frequency of recombination within genes to the genome average. Hugo Dooner, studying the *Bronze1* (*Bz1*) gene estimated that the ratio of genetic to physical distance within *Bz1* was 100-fold higher than the genome average (Dooner, 1986). This result was consistent with the conjecture that recombination is restricted to genes (Thuriaux, 1977), and stands in complete contrast to the initial view of recombination occurring only between genes.

## Crossing Over and Polarity

### Lessons From *S. cerevisiae* and *A. thaliana*

The foundation for our understanding of recombination is built upon intragenic recombination studies in fungal systems, particularly the budding yeast, *S. cerevisiae* (Nicolas and Petes, 1994; Gray and Cohen, 2016). These studies established a picture of recombination initiating at DSB hotspots which were usually found near TSSs (Petes, 2001). Later, this model was confirmed by genomic studies on the genome wide distribution of DSBs and meiotic recombination (Figure 1). DSB break maps, produced by capturing and sequencing SPO11-bound oligonucleotides released during initial DSB resection, confirmed that DSBs occur mainly near TSSs in *S. cerevisiae* (Pan et al., 2011). High resolution mapping of COs and NCOs placed 84% of recombination hotpots overlapping promoters near TSSs (Mancera et al., 2008). The agreement of genome-wide DSB maps and high-resolution recombination maps provides a clear picture of the general recombination pattern in *S. cerevisiae* (Table 2).

**Table 2:** Comparative characteristics of DSB hotspots.

	*S. cerevisiae*	*S. pombe*	*Mus musculus*	*Arabidopsis thaliana*	*Zea mays*
Number of DSB breaks/meiosis	∼160^1^	∼58^2^	230–350^3^	∼235^4^; 120^5^	∼500^6^
Number of DSB hotspots	3604^1^	603^2^	13,960^7^	5914^8^	3126^9^
	SPO11	SPO11 homolog	SPO11 homolog	SPO11 homolog	RAD51 homolog
DSBs mapped by:					
Hotspot width	189 bp median	965 bp median	143 bp median 99.8% < 2001 bp^7^	823 bp mean width^8^	1.2 kb; use of RAD51 may inflate hotspot width^9,10^
	73.4% between 50 and 300 bp, single peak^1^				
			Has secondary peaks^7^		
		range ∼50 bp – 7 kb^2^			
Location of DSBs	DSBs near TSS^1,11^	19% of DSBs within 200 bp of TSS^2,11^	3% of DSBs near TSS^11,12^	DSBs high at TSS and TTS^8,11^	DSBs high at TSS and TTS^9,11^
Importance of DSBs for COs	89% of DSBs in DSB hotspots. Recombination and DSBs are tightly linked^1^	72% of DSBs in hotspots. DSBs in cold regions account for almost half of COs ^2^	59.6% DSBs in DSB hotspots^7^. Account for ∼75% COs	Levels of DSBs and COs correlate at large scale, but no direct relation at fine scale^8^	Correlation of DSBs and COs only in genic regions^9^
DSBs in repetitive sequence?	Strongly under-represented^1^	Few^2^	Estimated 32.8% reads mapped to multiple sites^7^	Abundant, >50% of DSBs^8^	73.9% DSB in repetitive sequence^9^
Open chromatin, micrococcal nuclease sensitivity	DSBs mainly in NDR^1^ NDR provides access, other factors more important for determining if DSB occurs	DSBs are not concentrated in NDRs^2^	Most hotspots have a central NDR^8^	DSBs directly at NDRs^8^	Open chromatin^9^
H3K4me3	Association with hotspots may be indirect^12^	Low level of H3K4me3 at *ade6-M26* hotspot^13^	Presence at hotspots, serves to direct DSBs away from TSS^14^	Close to H3K4me3 sites at genes, but no correlation^8^	20% of all hotspots
					55% of genic hotspots^9^
DNA sequence motif				AT-richer^8^	G’s at 3 nt-periodicity^9^

*^1^Pan et al., 2011;*

*^2^Fowler et al., 2014;*

*^3^Plug et al., 1996;*

*^4^Chelysheva et al., 2007;*

*^5^Varas et al., 2015;*

*^6^Franklin et al., 1999;*

*^7^Lange et al., 2016;*

*^8^Choi et al., 2018;*

*^9^He et al., 2017;*

*^10^DSB mapping with SPO11 provides higher precision than with RAD51 (Pan et al., 2011);*

*^11^TSS, transcription start site and TTS, transcription termination site;*

*^12^Tischfield and Keeney, 2012;*

*^13^Yamada et al., 2013; ^14^Brick et al., 2012.*

In Arabidopsis, DSB hotspots also localize to gene promoters, additionally to terminators, as well as introns (Choi et al., 2018). Though only a fraction of DSBs is resolved into COs in Arabidopsis, DSB and CO levels were shown to correlate strongly at the chromosome scale, though varying along arms (Choi et al., 2018) (Table 2).

The recombination machinery has been extensively described and reviewed in general as well as in plants (Pradillo et al., 2014; Lambing et al., 2017). Briefly, recombination initiates with a DSB. Resection creates a 3′-overhang that invades a homologous DNA region and pairs with its complementary sequence, binding it as a repair template. DNA synthesis can then proceed from the exposed 3′-end. From here, the invading strand plus newly synthesized sequence may dissociate from the complementary strand giving non-crossover events through SDSA (synthesis-dependent strand annealing). Alternatively, a double Holliday junction structure may form which can resolve into a crossover.

Both COs and NCOs give rise to a short region with non-reciprocal transfer of genetic information known as a gene conversion tract. The length of gene conversion tracts depends on DSB resection, synthesis from the exposed 3′-end, and migration of Holliday junctions. Median gene conversion tract lengths in *S. cerevisiae* have been measured at 2.0 kb for COs and 1.8 kb for NCOs (Mancera et al., 2008). NCO conversion tracts reached up to 40.8 kb in length, but 97% were less than 5 kb in length. Some CO and NCO conversion tracts had complex tracts arising via template switching between the parental alleles (Marsolier-Kergoat et al., 2018). In general, crossovers in *S. cerevisiae* are located close to the position of initiating DSBs.

Gene conversion tracts in Arabidopsis wild type have been far more difficult to detect and characterize, and are in general fewer and shorter than in budding yeast. For COs, gene conversion tracts were detected first at a maximal median length of ∼1.1 kb, for NCOs in the range of 1 bp to ∼6.6 kb (Lu et al., 2012). In another study, Arabidopsis NCO gene conversion ranged from mean tracts of 1 bp to ∼0.5 kb, the longest at ∼3.0 kb (Drouaud et al., 2013). The marker resolution underlies the precision at which gene conversion tracts can be defined, and might explain the even shorter estimates of CO-associated tracts of ∼0.3–0.4 kb and NCO-associated tracts of 25–50 bp (Wijnker et al., 2013).

Polarity for recombination is seen in many *S. cerevisiae* genes. The small discrete DSB hotspots located near TSSs concentrate recombination events at the 5′ end of many genes. A DSB hotspot at the 5′ end of a gene can give polarity near the 3′ end of a neighboring gene. Variation in gene conversion tract length and mismatch repair both contribute to polarity (Nicolas and Petes, 1994). Polarity can also be seen in Arabidopsis genes where recombination peaks near TSS, then decreases toward the end of genes (Hellsten et al., 2013; Choi et al., 2016). In maize, the relative importance of polarity in recombination is one of the questions arising between intragenic recombination studies and high-throughput genotyping studies.

### Distribution of Crossovers in Maize Intragenic Studies

In a series of studies beginning in 1985, and continuing today, Hugo Dooner described a number of properties of maize recombination using the *Bz1* locus (Table 1). For genetic crossovers, there is no polarity within *Bz1*. The ratio of physical distance to genetic distance (kb/cM) at the 5′ and 3′ ends of *Bz1* are similar (Dooner and Martínez-Férez, 1997). This absence of polarity extends beyond *Bz1* into adjacent sequences. Upstream, the genetic distance from a marker within *Bz1* to the upstream gene *Mkk1* 100 kb away was less than the genetic length of *Bz1* (Dooner, 1986; Fu et al., 2002). Similarly, no crossovers were detected in the downstream interval between *Bz1* and the adjacent gene, *Stc1* (He and Dooner, 2009). There is, however, polarity for NCOs at both ends of the *Bz1* gene. Point mutations at both ends of *Bz1* are converted more frequently than point mutations in the middle of the gene. 5′ flanking sequences are required for polarity at the 5′ end; the requirement for 3′ flanking sequences have not been tested (Dooner and He, 2014). To summarize, the number of NCOs peak at the ends of *Bz1*, but COs are evenly distributed across the entire *Bz1* coding region and are rare in upstream and downstream regions.

Outside of *Bz1* the most extensive intragenic recombination data comes from the *Wx* locus. Here too there is no evidence for polarity of COs. Nelson fine-mapped 29 *wx* alleles, and a number of these mutations were sequenced in Sue Wessler’s lab. Looking at recombination between pairs of alleles with a variety of genetic backgrounds and mutational lesions found no indication of polarity at *Wx* (Okagaki and Weil, 1997). Though limited to only four alleles, results at the rice *Wx* locus are in agreement (Inukai et al., 2000). Similarly, at the maize *Stc1* locus, COs are found across the length of the gene with similar numbers of crossovers at the 5′ and 3′ ends of the gene (Dooner and He, 2008; He and Dooner, 2009).

Contrasting with the absence of polarity at the *Wx* locus is the strong polarity for COs at the maize *A1*, *B1*, and *R1* genes. 5′-polarity was seen at *A1* and *B1*. Thirty-three of 35 crossovers in *B1* mapped to a 620 bp interval overlapping the start codon (Patterson et al., 1995. The *A1* gene has a 377 bp hotspot beginning in exon 1 and spanning exon 2 (Xu et al., 1995). Recombination at *R1* showed a polarity gradient with highest levels of recombination at the 3′-end of *R1* that declined to low levels in the middle of the gene, a distance of approximately 3.5 kb (Eggleston et al., 1995; Dietrich, 1998; Kermicle, personal communication).

Intragenic recombination studies have also looked at recombination within small genetic intervals. Since high recombination rates measured within genes suggests that little recombination happens in intergenic regions this work directly asks whether crossing over can take place outside of genes. Studying recombination in the *Al – Sh2* interval, Patrick Schnable’s group identified three CO hotspots in the 130–140 kb interval (Yao et al., 2002; Yao and Schnable, 2005). Two of the four genes in the region were CO hotspots, and the third hotspot was in a unique non-genic sequence. Only four of the 101 COs mapped outside of the three hotspots (Yao et al., 2002). The genic region surrounding *Bz1* presents a similar pattern with a majority of the genes in the region functioning as CO hotspots (Fu et al., 2002; He and Dooner, 2009). The large block of repetitive sequence upstream of *Bz1* is heavily methylated consistent with methylation suppressing recombination as has been seen in Arabidopsis (Melamed-Bessudo and Levy, 2012; Mirouze et al., 2012; Yelina et al., 2015). Haplotype structure and local sequence differences locally suppressing recombination provides an additional mechanism for modifying crossover frequencies (Yao and Schnable, 2005; Dooner and He, 2008).

In summary, the key results from intragenic recombination studies in maize are as follows: (1) both crossover and non-crossover events are detected; (2) many maize genes are recombination hotspots; (3) some but not all genes show polarity that may be punctate or have a gradient; (4) some recombination hotspots are in non-genic low-copy sequences; (5) sequence differences between parental chromosomes affect the distribution of recombination events. However, the number of studies with adequate data is small and conclusions about relative frequency of genes showing polarity should not be drawn.

### Distribution of Maize Crossovers by High-Throughput Genotyping

At the genome-wide scale, maize COs form a particular U-shape pattern, with COs increasing strongly toward chromosome ends (Anderson et al., 2003; Li et al., 2015; Rodgers-Melnick et al., 2015; Kianian et al., 2018). Maize chromosomes have rather big pericentromeric heterochromatin regions that cover more than half of them (Baucom et al., 2009; Wei et al., 2009). Heterochromatin is thus negatively correlated with COs at large scale, but we want to keep the focus on the gene-scale data to allow comparison between traditional intragenic studies in maize and the newer cohort of sequencing-technology-driven studies.

Four studies based on next-generation sequencing have mapped recombination events in maize (Table 3). Three studies have been published (Li et al., 2015; Rodgers-Melnick et al., 2015; Kianian et al., 2018). Data from the fourth study was reported in Alina Ott’s Ph.D. dissertation, and a manuscript is in preparation (Ott, 2017). High marker density is critical for studying polarity and other questions. Three maize genome-wide studies reported polarity for crossovers, with COs most frequent in the 5′ region of genes and low in the central region of genes (Li et al., 2015; Ott, 2017; Kianian et al., 2018). The resolution of one study was generally not sufficient to address this question (Rodgers-Melnick et al., 2015). Crossovers were mapped with sufficient precision to identify polarity for approximately 50% (Kianian et al., 2018) and approximately 10% of crossovers placed (Li et al., 2015; Ott, 2017). Two of the studies reported evidence for high crossover frequency at the 3′ end of the gene (Li et al., 2015; Kianian et al., 2018). Recombination polarity at the 5′ and often 3′ ends of genes has been reported to be the common pattern in several plant species (reviewed in Choi and Henderson, 2015).

**Table 3:** Maize sequencing based CO studies.

Study	Li et al., 2015	Rodgers-Melnick et al., 2015	Kianian et al., 2018	Ott, 2017
Crossover measurement	DNA-seq of tetrads after WGA	GBS of RILs	DNA-seq of backcrossed F1 plants	RNA-seq of RILs
Coverage	Low (∼1.4x)		Low (∼1.5–5x)	
COs per meiosis	19.2	Most between 20 and 25	17.2 (male), 18.6 (female)	
Marker density	Median 1 SNP/ 235 bp		Median 1 SNP/44 bp	1.3 SNPs/kb of gene
Number of individuals	96 (24 tetrads)	4714 (US-NAM), 1382 (China-NAM)	135 (male), 122 female	105
Crossover intervals	∼63% ≤ 100 kb	Median 127 kb (10% ≤ 10 kb)	∼50% ≤ 2 kb	Median 104.6 kb
Hotspot definition	n.d.	Regions containing a concentration of narrow crossover intervals. Estimated FDR of 0.5%	5 kb region with ≥5x genome average	Genes with ≥2 crossovers
Number of crossovers	924	103,459 (US-NAM)	1164 (male)	7574
			1139 (female)	
		32,536 (China-NAM)		
Number of crossovers mapped short interval	234 ≤ 10 kb	10% ≤ 10 kb	∼50% ≤ 2 kb	793 mapped within a gene
Number of hotspots	n.d.	410	282 (male)	158
			257 (female)	
Percent of crossovers outside of hotspots	n.d.	Estimated 70%	n.d.	n.d.
Percent of genome with crossover hotspot	n.d.	≤0.2%	∼0.05%	n.d.

Crossover hotspots were identified in three studies (Rodgers-Melnick et al., 2015; Ott, 2017; Kianian et al., 2018). Kianian’s study defined hotspots as 5 kb regions with CO rates fivefold higher than the genome average; there were 282 and 257 hotspots in the male and female parents of the population respectively (Kianian et al., 2018). Using the 793 COs mapped within a gene, Ott identified 158 genes with more than one CO event in her population; many of these genes are statistically likely to be CO hotspots (Ott, 2017). These two studies relied on relatively small populations. Using a much larger population, Rodgers-Melnick’s study found 410 hotspots (Rodgers-Melnick et al., 2015). Not all of the genic hotspots described by intragenic recombination studies were identified in these studies. Sampling depth may be a limiting factor in detecting CO hotspots, but the variable number and locations of CO hotspots suggests we need to think carefully about the meaning of hotspots.

**FIGURE 3:**
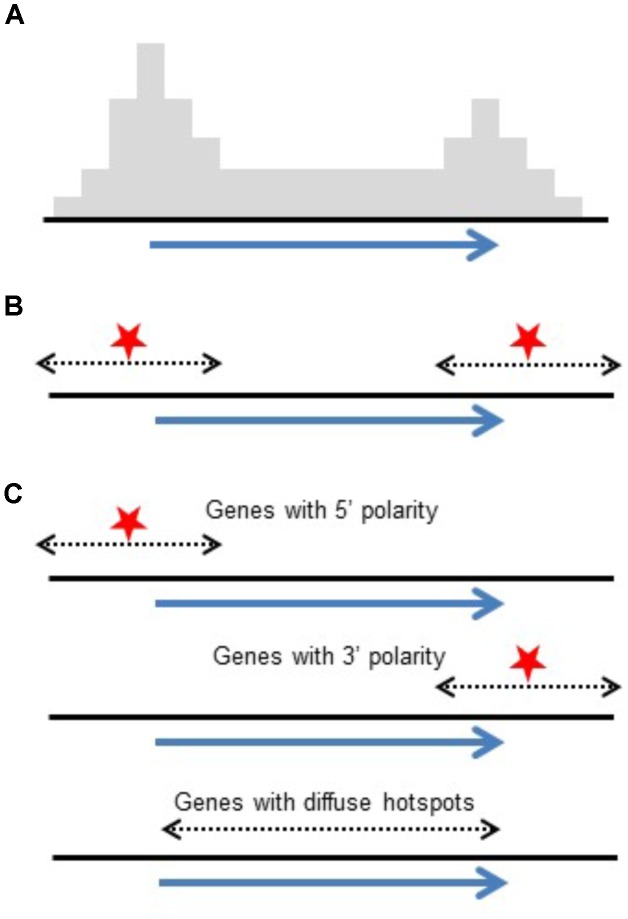
Reconciling polarity gradients as seen in high-throughput genotyping versus intragenic studies. **(A)** Histogram representing crossover polarity seen in high-throughput genotyping studies. **(B)** DSB hotspots at both ends of genes could produce this distribution. **(C)** Alternatively, a mix of genes having DSB hotspots at their 5′ ends, their 3′ ends, and genes that are diffuse hotspots give the same pattern of crossovers.

Conclusions drawn from high-throughput genotyping studies emphasized polarity with COs concentrated at 5′ and 3′ ends of genes (Li et al., 2015; Ott, 2017; Kianian et al., 2018). Although this differs at first glance from intragenic studies which reported genes with and without polarity, the data actually agrees. Intragenic studies reporting on individual genes found a mix of genes showing polarity and others that do not. What is reported in genome-wide studies is an accumulated pattern from 100s of genes. The polarity found in these studies could be a result of a generalized polarity at most genes or the result of a mix of genes with and without CO polarity as seen in intragenic studies. While high-throughput studies emphasize increased CO rates at 5′ and 3′ ends of genes, intragenic studies report on individual genes, exposing the mix of genes with large diffuse hotspots or localized hotspots (Figure 3). Similarly, in Arabidopsis, it has been shown that the level of polarity-underlying DSBs at the TSS and TTS are independent from each other (Choi et al., 2018).

## Double-Strand Break Hotspots

### Lessons From *S. cerevisiae* and *A. thaliana*

In *S. cerevisiae*, most meiotic DSBs resolve into COs and NCOs. The 140–170 DSBs observed per yeast meiosis (Buhler et al., 2007) closely match the 90.5 COs and 46.5 NCOs counted per meiosis (Mancera et al., 2008). Precise mapping of DSB hotspots via SPO11-bound nucleotides released at resection revealed that almost 90% of DSBs occurred in a described hotspot (Pan et al., 2011) (Table 2). DSBs were underrepresented in repetitive sequences with repetitive DNA comprising 14% of the genome while accounting for only 1.16% of DSB breaks defined by SPO11 reads (Pan et al., 2011). Overall, 95% of DSBs identified by SPO11 oligonucleotides were confined to just 15% of the yeast genome (Marsolier-Kergoat et al., 2018). In Arabidopsis, mapping of SPO11-oligonucleotides also revealed that DSBs are preferentially located in regions with high gene density, and underrepresented in TE dense regions (Choi et al., 2018).

Although there was no consensus target sequence for the SPO11 nuclease responsible for meiotic DSBs, the DNA sequence was non-random with preferred nucleotides at certain positions, and a central AT-rich sequence surrounded by modestly GC-rich sequence. AT-rich motifs were also found at Arabidopsis DSB sites (Choi et al., 2018), likely due to those motifs generally excluding nucleosomes (Segal and Widom, 2009).

*Saccharomyces cerevisiae* DSBs map preferentially to nucleosome depleted regions (NDRs) around the TSS, a hallmark of open chromatin (Pan et al., 2011). The trimethylated histone H3 lysine 4 (H3K4me3) is a histone modification promoting an open chromatin structure that is found at DSB hotspots (Borde et al., 2009). Noteworthy, however, is that not all NDRs are DSB hotspots and H3K4me3 is absent at some DSB hotspots (Pan et al., 2011; Tischfield and Keeney, 2012). This picture of open chromatin being favored by the DSB machinery is also true in Arabidopsis: here, DSBs were shown to correlate with H3K4me3 and low nucleosome density (Choi et al., 2018).

In summary, the correlation of DSBs and recombination in *S. cerevisiae* is clear as almost all DSBs lead to recombination and both the initiating DSBs and resolving recombination hotspots are mainly found near TSSs. The picture is different in other systems, including Arabidopsis, where a few 100 DSBs get resolved into only ∼6–12 COs (Giraut et al., 2011; Lu et al., 2012; Salomé et al., 2012; see Table 2).

### Location and Distribution of Maize Double-Strand Breaks

Double-strand breaks cannot be directly mapped by intragenic studies, but their possible positions may be deduced by examining recombination in deletion mutations. Homozygous deletions of DSB hotspots reduce recombination and eliminate polarity at the *S. cerevisiae HIS4* locus (Detloff et al., 1992). The maize *wx-B* allele is a 1 kb deletion around the TSS from -459 to +505, while *wx-C4* has a smaller deletion within the transcribed region from +257 to +454 (Wessler et al., 1990; Okagaki and Weil, 1997). If DSBs near the TSS contribute substantially to recombination, then more recombinants should be recovered between alleles with intact TSS regions than with alleles containing TSS region deletions. This, however, was not seen when Oliver Nelson measured recombination between *wx-B* and *wx-C4* with downstream alleles (Supplementary Table 1). Second, there is directionality to the repair of meiotic DSBs since DSBs are repaired using sequences on the homologous chromosome. Thus in a line where one allele contains a deletion of its DSB hotspot and the other allele retains its DSB hotspot, recombination preferentially deletes the previously non-mutant sequence (Nicolas et al., 1989). The *wx-B1* allele has a deletion from -655 to +299 (Wessler et al., 1990). Recombination between *wx-B1* and the *wx-I* allele, containing a large insertion in the 3′ region, also argued against a single DSB hotspot near the TSS (Okagaki and Weil, 1997). Here, 28 of 29 recombinants were crossovers between *wx-B1* and *wx-I*. The DSBs in this experiment most likely were in the region between *wx-B1* and *wx-I* rather than in the 5′ region.

Indirect evidence against a yeast-like concentration of DSBs near TSSs in maize comes from one whole genome study. A high-throughput recombination study via the maize transcriptome mapped 2634 NCO conversion tracts in a maize RIL population (Ott, 2017). Non-crossover conversion tracts in the 5′, the central, and 3′ regions of maize genes were roughly equally distributed, with a slightly higher number in the middle region of genes (Ott, 2017). According to the original canonical DSB model, gene conversion tracts produced at NCOs and COs will flank or encompass the position of initiating DSBs, thus indirectly mapping DSBs (Szostak et al., 1983). More recent data in *S. cerevisiae* shows that NCO conversion tracts can be located a short distance from the DSB (Marsolier-Kergoat et al., 2018). The even distribution of NCO conversion tracts across maize genes argues against the concentration of DSBs at the ends of maize genes.

A recent maize genomic DSB study using a genome-wide approach similar to the one used in *S. cerevisiae* and other model species captured and sequenced single-stranded DNA bound to RAD51 (He et al., 2017). In total, the maize DSB mapping effort identified 3126 hotspots (He et al., 2017), similar to the 3604 DSB hotspots in *S. cerevisiae* (Pan et al., 2011). The number of defined maize DSB hotspots is conservative. With relaxed stringency and controls the number is considerably larger (He et al., 2017). Although maize DSB hotspots shared some characteristics with their *S. cerevisiae* counterparts and DSB hotspots from Arabidopsis and other model systems, there are some clear differences (Table 2). The almost 73% of maize DSB hotspots in repetitive sequence contrasts with the under-representation of DSB hotspots in *S. cerevisiae* repetitive sequences. Maize DSB hotspots were also wider than *S. cerevisiae* DSB hotspots, 1.2 kb versus 189 bp. This difference might be a consequence of the precision of the SPO11 based approach used in *S. cerevisiae* versus the lower resolution possible with the RAD51 based approach used in maize, or it could represent physical differences in their DSB hotspots. Perhaps of greater interest is the close relationship between DSB hotspots and COs found in *S. cerevisiae* may not hold in maize (see section “The Role of Double-Strand Breaks in Maize and Other Model Organisms”). One mapped DSB hotspot was immediately upstream of *Bz1*. However, as we have seen from intragenic studies, COs at *Bz1* do not show polarity, although NCOs show polarity at the 5′ and 3′ ends of *Bz1* (Dooner and Martínez-Férez, 1997; Dooner and He, 2014). Thus the importance of a DSB hotspot flanking *Bz1* for recombination is unclear.

Approximately, 85% of the maize genome is composed of families of repetitive elements widely distributed in the maize genome (Schnable et al., 2009). Ty1-gypsy-like elements are the most abundant families (Meyers et al., 2001). Over 50% of maize DSB hotspots are in gypsy-like elements (He et al., 2017). Both DNA methylation and sequence polymorphisms are suggested mechanisms for suppressing COs in maize repetitive sequences (Fu et al., 2002; Yao and Schnable, 2005). Repetitive elements are commonly found in blocks separating individual genes or small clusters of genes (Haberer et al., 2005). These blocks are not conserved between maize lines, and two genes may be separated by a short stretch of low-copy sequence in one line and by significant stretches of repetitive sequence resulting from multiple repetitive elements in another line (He and Dooner, 2009). This absence of sequence homology will strongly inhibit recombination. Even when the overall structure of a repetitive block is preserved, nucleotide polymorphisms could locally inhibit crossing over, as seen in the a1-sh2 region (Yao and Schnable, 2005). However, results from one intragenic recombination study argues against sequence polymorphism as the primary mechanism suppressing crossing over in repetitive sequences (Fu et al., 2002). In this study using the Bz1 region, crossing over was compared between a short genic region and a block of repetitive sequence flanking the genes. Because the haplotypes used in this experiment were derived from the same progenitor, there was little if any sequence difference in the region except for the genetic markers. In this region, at least, sequence differences cannot account for the lack of crossing over in repetitive sequence.

### The Role of Double-Strand Breaks in Maize and Other Model Organisms

Meiotic DSBs serve two functions, first to promote chromosome pairing and second to produce the crossovers necessary to ensure proper segregation of chromosomes at anaphase (Page and Hawley, 2003). Meiotic DSBs can be visualized on chromosomes as RAD51 foci (Franklin et al., 1999). In mid-zygotene when chromosomes are pairing, there are approximately 500 RAD51 foci decreasing to about 12 RAD51 foci in pachytene (Franklin et al., 1999; Pawlowski et al., 2003). The zygotene foci are distributed across the chromosomes where single-stranded DNA ends produced by DSBs and resection promote chromosome alignment (Smithies and Powers, 1986; Peoples-Holst and Burgess, 2005). The pachytene foci, also known as late recombination nodules when viewed with electron microscopy, represent the sites of crossing-over (Stack and Anderson, 2002). Control of the number of COs and which DSBs are channeled into the crossover pathway is tightly regulated (Lake and Hawley, 2016). There are two types of COs, with interference-sensitive COs (type I) constituting the majority of COs, and a few additionally interspersed interference-insensitive COs (type II) in many species (Gray and Cohen, 2016). Some species lack the type I interference-sensitive pathway (Gray and Cohen, 2016). Though their mechanisms are distinct, their outcomes are treated equally in intragenic as well as whole genome studies.

Double-strand breaks are necessary for recombination, but the importance of DSB hotspots for genetic crossovers is less clear. Compared with *S. cerevisiae*, the fraction of maize DSBs resolving as crossovers is small. Mapping crossovers using *S. cerevisiae* tetrads gave an average of 90.5 crossovers per meiosis, with an estimated 160 DSBs per meiosis (Mancera et al., 2008; Pan et al., 2011). In contrast, only a fraction (∼5–10%) of DSBs get resolved into COs in Arabidopsis (Giraut et al., 2011; Lu et al., 2012; Salomé et al., 2012; also see Table 2). Mapping crossovers from maize tetrads determined an average of 19 crossovers per meiosis (Li et al., 2015), similar to the range of cytologically determined CO in maize inbreeds (Sidhu et al., 2015). Thus, less than 4% of maize DSBs resulted in COs versus 56% in *S. cerevisiae* tetrads. In fact, the majority of DSB hotspots appear unlikely to contribute much to crossing over as almost 73% of hotspots are in repetitive sequence where crossovers are believed to be suppressed (He et al., 2017). It seems reasonable to conclude that a majority of maize DSBs promote chromosome alignment (Peoples-Holst and Burgess, 2005).

The genome-wide maize DSB data identified 3126 high-confidence DSB hotspots, about one-fourth of them in genes. This is a conservative estimate of the number of hotspots based on very stringent criteria (He et al., 2017). What the genomic maize DSB study shows clearly, however, is the concentration of DSBs around TSS and TTS of many genes (He et al., 2017). This is mirrored in other model systems, for example Arabidopsis, with highest DSB levels at TSS and TTS (Choi et al., 2018), and is in general a prerequisite for recombination polarity along a gene body.

Though the concept is enticing, enrichment of maize DSB hotspots around the TSS and TTS of genes may not exist at all genes, with or without producing polarity. On average, DSBs have an increased tendency to peak at TSS and on both sides of TTS (He et al., 2017), agreeing with CO peaks close to TSS and TTS (Kianian et al., 2018). However, polarity for CO is not seen at *Bz1* despite the adjacent DSB hotspot (Dooner and Martínez-Férez, 1997; He et al., 2017). In *S. cerevisiae*, the tight connection between DSB hotspots and recombination is well-established (Pan et al., 2011; Marsolier-Kergoat et al., 2018), but there are large differences between eukaryotes (Fowler et al., 2014; Stapley et al., 2017). Results from similar studies in the fission yeast *Schizosaccharomyces pombe* (*S. pombe*) present a very different picture (Table 2). Less than 20% of *S. pombe* DSB hotspots are near the TSS. Furthermore, DSBs in *S. pombe* hotspots are preferentially repaired from the sister chromatid; these events do not contribute to crossing-over. A large fraction of COs are initiated at non-hotspot DSBs in *S. pombe* (Fowler et al., 2014).

On the other hand, *S. pombe* DSB hotspots are not strongly correlated with NDRs (Fowler et al., 2014). This contrasts with *S. cerevisiae*, Arabidopsis and maize.

Mouse DSB hotspots share characteristics of both *S. cerevisiae* and *S. pombe* and hint at the complexities underlying hotspots. DSB hotspot widths are similar to *S. cerevisiae* (Lange et al., 2016). Unlike *S. cerevisiae*, these hotspots are over-represented within the genic region defined by the start and stop codons, and only 3% are located near the TSS (Smagulova et al., 2011; Brick et al., 2012). The H3K4me3 modification at mouse DSB hotspots is produced by the *Prdm9* histone methyltransferase (Baudat et al., 2010). H3K4me3 is present at other sites along mouse chromosomes including TSS. Removing H3K4me3 at DSB hotspots using *prdm9* mutant mice blocks DSBs from forming at these hotspots. Instead, DSBs occur at other H3K4me3 sites on the chromosome; many of these are near the TSS (Brick et al., 2012). Of critical importance here is the observation that these mice are defective in DSB repair and chromosome pairing (Hayashi et al., 2005).

Open chromatin is so far the only universal criteria for DSBs and CO locations across different model systems, though caution is needed regarding the scale (Tischfield and Keeney, 2012). The active chromatin mark H3K4me3 for example can be found near DSBs, but overlaps COs even more, arguing that it promotes recombination downstream of DSBs, in yeast and Arabidopsis, while DSB association is merely due to location to promoters (Tischfield and Keeney, 2012; Choi et al., 2018). Rather, chromosomal context and the relationship between the DSB and the synaptonemal complex are important (Medhi et al., 2016). DNA methylation is yet another component underlying the structure of the chromosome, but details on its association with DSB and COs are beyond the scope of this review, which focused on intragenic recombination.

## No Model System Explains All

As described here, there are aspects of systems other than *S. cerevisiae* that provide insight into maize. For example, in *S. pombe*, DSBs in hotspots contribute far less to COs than expected – most DSBs in hotspots do not resolve as COs. Might this be similar to maize where three-fourths of DSB hotspots are in repetitive sequence? Does the absence of class I COs in *S. pombe* disqualify *S. pombe* as a model system for maize recombination, any more than the under-representation of DSB hotspots in *S. cerevisiae* repetitive sequence disqualifies *S. cerevisiae* as a model for maize where three-fourths of DSB hotspots are in repetitive sequence? Is there a clear reason why class I versus class II COs are a more important criteria for choosing a reference model system than the weak association between DSB hotspots and COs in maize and *S. pombe* versus the tight association in *S. cerevisiae*?

Deleting the promoter region in a yeast gene strongly reduces recombination at the gene (De Massy and Nicolas, 1993; Porter et al., 1993). Deleting the promoter region in maize *Bz1* and *Wx* does not strongly reduce recombination at the gene (see section “Lessons From *S. cerevisiae* and *A. thaliana*,” Supplementary Table 1).

Mouse provides the valuable lesson that open chromatin in promoters is not necessarily a target for DSBs. On the other hand, mouse DSB hotspots are mediated by PRDM9 which does not seem to exist in plants. Are plants as the mostly related species not the best system to compare with maize? In spite of different genome architecture of Arabidopsis and maize, many commonalities of DSB and CO hotspots can indeed be found. The agreement of recombination distribution is even better when comparing other large-genome crops with each other, as for example maize, wheat, and barley (Higgins et al., 2012; Darrier et al., 2017). Only when integrating information learned from different model organisms and different approaches do we have a chance of resolving the whole story on DSBs, recombination, polarity, and underlying genome features.

## Summary

Maize has been a genetic model genetic system for almost 100 years, and has been used to address questions regarding recombination as fundamental as the connection between cytological crossing over and genetic crossing over (Creighton and McClintock, 1931). However, there is a chasm between what we know based on extensive data, what we think we know, and what is known in other model systems used for studying recombination. Intragenic studies on small genetic regions have characterized most genes as recombination hotspots, but some genes are coldspots and some non-genic regions are recombination hotspots (Yao et al., 2002; He and Dooner, 2009; Wang et al., 2011). Issues may arise when extrapolating results from the handful of maize genes where intragenic recombination has been studied in depth. On the other hand, high-throughput genotyping studies suffer from a lack of resolution or depth, and small sample sizes. The median interval defining crossovers in three of the four studies was over 100 kb (Li et al., 2015; Rodgers-Melnick et al., 2015; Ott, 2017). Greater precision is necessary to minimize the misleading correlations possible with low-resolution mapping (Tischfield and Keeney, 2012).

Where do crossovers occur in maize? At the chromosomal level, intragenic and genome-wide studies identified an association between gene-density and elevated crossover rates (Yao et al., 2002; Wang et al., 2011; Pan et al., 2017). Looking at single genes or small genetic intervals, intragenic studies conclude that most crossovers take place within genes. Genes appear to fall into two categories with crossovers either concentrated at one end of the gene, either 5′ or 3′ polarity, or distributed evenly across the gene (Eggleston et al., 1995; Patterson et al., 1995; Dooner and Martínez-Férez, 1997; Okagaki and Weil, 1997; Yao and Schnable, 2005). High-throughput genotyping studies, drawing parallels with the polarity found in *S. cerevisiae* genes, emphasize polarity at the 5′ and 3′ ends of genes (Li et al., 2015; Ott, 2017; Kianian et al., 2018). But two of these studies determined that about one-fourth of the crossovers mapped within a gene were in the central region (Li et al., 2015; Ott, 2017). In the third study, 50% of the crossovers did not map to either the 5′ or 3′ regions (Kianian et al., 2018). Intragenic studies look at many recombination events at a few genes while genomic approaches average over many genes with few recombination events each. The two approaches give different snapshots and interpretations of crossing over and point to their relative strengths and weaknesses.

Due to the underlying literature we have focused this review on hotspots for crossover and DSBs. This perspective may be problematic. It seems that hotspots are not the best means to describe crossovers in maize which could be better described as following a chromosome-wide polarity gradient toward the chromosome ends coupled with an avoidance of repetitive sequence in the case of COs. This wider perspective encompasses additional questions including the importance of trans-acting modifiers, frequently genes from recombination pathways (Pan et al., 2017).

In addition, the fraction of crossovers attributed to recombination hotspots is not high. The 410 CO hotspots defined by Rodgers-Melnick accounted for 30.6% crossovers defined to a narrow interval (Rodgers-Melnick et al., 2015). Our interest in hotspots seems to be focusing our attention on local determinants of recombination as a functional unity and away from the larger and possibly more important chromosomal context (Pan et al., 2011). Where a DSB occurs along a chromosome may be just as important in determining the DSB fate as local hotspot features (Serrentino and Borde, 2012). There is now an intense interest and effort in understanding the roles of chromatin features including the synaptonemal axis, loops, and cohesin proteins, which will help refine our views on meiotic recombination mechanisms and patterns (Barrington et al., 2017).

## Author’s Note

Results from Alina Ott’s Ph.D. dissertation have now been published in Liu et al. (2018).

## Author Contributions

RO: conception of this project. SD-S: re-analyzing recombination data. WE: *R1* recombination data. RO and SD-S: drafting of manuscript. RO, SD-S, WBE, and GM: editing of manuscript. All authors approved the final version of the manuscript.

## Conflict of Interest Statement

The authors declare that the research was conducted in the absence of any commercial or financial relationships that could be construed as a potential conflict of interest.
