# Supplementary material for: A Critical Assessment of 60 Years of Maize Intragenic Recombination
Source: Front Plant Sci. 2018 Oct 29;9:1560. doi: 10.3389/fpls.2018.01560 (PMC6215864; doi:10.3389/fpls.2018.01560)
Supplement: Supplementary file 1 [file Table_1.docx]

#### Supplemental Table 1: Pollen reversion frequencies comparing recombination with a deletion including the promoter (*wx-B*) against an overlapping deletion that does not extend into the promoter (*wx-C4*)^1^

| Deletion allele | x *wx-m1*^2^ | x *wx-C*^2^ | x *wx-I*^2^ |
| --- | --- | --- | --- |
| *wx-B* (-459 to +505) | 41 +/- 1.6^3^ | 45 +/- 10.6^3^ | 58 +/- 3.2^3^ |
| *wx-C4* (+257 to +454) | 47 +/- 4.3^3^ | 57 +/- 3.9^3^ | 35 +/- 2.1^3^ |

^1^ Data from Nelson, 1968

^2^ Deletion allele crossed with: *wx-m1*, a *Ds* insertion in exon 9; *wx-C*, a deletion in exon 7; and *wx-I*, a retrotransposon insertion in exon 12.

^3^ Revertant pollen grains per 100,000 grains
